# The optimal timing of additional surgery after non-curative endoscopic resection to treat early gastric cancer: long-term follow-up study

**DOI:** 10.1038/s41598-019-54778-8

**Published:** 2019-12-04

**Authors:** Jae Hwang Cha, Jie-Hyun Kim, Hyoung-Il Kim, Da Hyun Jung, Jae Jun Park, Young Hoon Youn, Hyojin Park, Seung Ho Choi, Jae-Ho Cheong, Woo Jin Hyung, Sung Hoon Noh

**Affiliations:** 1 Department of Internal Medicine, Yonsei University College of Medicine, Seoul, Korea; 2 Department of Surgery, Yonsei University College of Medicine, Seoul, Korea; 3 Gangnam Severance Hospital, Yonsei University College of Medicine, Seoul, Korea; 4 Department of Surgery, Severance Hospital, Yonsei University College of Medicine, Seoul, Korea; 5 Department of Internal medicine, Dong-A University College of Medicine, Busan, Korea

**Keywords:** Gastric cancer, Stomach

## Abstract

Patients with early gastric cancer (EGC) who undergo non-curative endoscopic resection (ER) require additional surgery. The aim of the study was to validate surgical and oncological outcomes according to the timing of additional surgery after non-curative endoscopic resection. We retrospectively analyzed long-term follow-up data on the 302 patients enrolled between January 2007 and December 2014. We validated our earlier suggestion that the optimal time interval from non-curative ER to additional surgery was 29 days. All patients were divided into two groups by reference to time intervals from ER to additional surgery of ≤29days (n = 133; group A) and >29 days (n = 169; group B). The median follow-up duration was 41.98 ± 21.23 months. As in our previous study, group B exhibited better surgical outcomes. A total of 10 patients developed locoregional or distant recurrences during the follow-up period, but no significant difference was evident between the two groups. Interestingly, the survival rate was better in group B. Group B (>29 days) exhibited better surgical and oncological outcomes. Thus, additional gastrectomy after non-curative ER should be delayed for 1 month to ensure optimal surgical and oncological outcomes.

## Introduction

Gastric cancer (GC) is a major health problem worldwide, with an estimated 1 million new cases per year^1^. Early gastric cancer (EGC) is defined as a lesion confined to the mucosa or the submucosa, regardless of the presence of regional lymph node metastasis (LNM), and some of them can be cured via endoscopic resection (ER)^2–4^. ER for EGC plays a central role in the treatment of EGC, and due to the development of the endoscopic technology and instrument, extended criteria are being applied beyond the absolute indication for ER, nowadays^5–7^. Although ER has the advantage of preserving the stomach, is minimally invasive, and affords a better quality of life than open surgery, ER sometimes fails to completely remove a lesion^8^. The rates of non-curative ER range from 15.3 to 16.7%^9–11^. Patients with non-curative ER typically require additional treatment such as re-ER, ablation therapy, and/or gastrectomy^12–14^. However, additional surgical gastrectomy with lymph node dissection is generally recommended after non-curative ER because of the possibility of LNM and the unfavorable prognosis^7,13,15,16^. However, the optimal timing of additional surgery after non-curative ER remains unclear. In our previous study, we evaluated the time interval between ER and additional surgery in terms of surgical and oncological outcomes^17^. We found that the interval affected the surgical outcomes^17^.

ER-associated electrocoagulation creates a large iatrogenic ulcer requiring 4–8 weeks for complete healing. Also, ER may cause edema, fibrosis, and even adhesions of both the stomach and surrounding tissues, which may be increasing the surgical difficulties during subsequent gastrectomy^18,19^. However, waiting for the healing of edema or ulceration after ER in cancer patients may allow for tumor to grow and increase the risk of recurrence. Another essential aspect that must also be taken to account is the potential impact of treatment delay on patient anxiety. Although patients know that it is necessary to wait for a treatment, it is experienced as a suffering time of anxiety and fear. Many surgeons plan the timing of operation after non-curative ER by reference to their surgical schedules or other subjective factors. It is important to objectively evaluate the optimal effect of the time interval of additional gastrectomy after ER on surgical outcome.

Our previous study suggested an optimal time interval between non-curative ER and additional surgery; however, the work had certain limitations, including a relatively small number of patients and only short-term follow-up. We thus could not confirm the oncological outcomes. Here, we analyze long-term follow-up data to validate the surgical outcomes and evaluate the oncological outcomes associated with our previously proposed optimal time interval from non-curative ER to additional surgery.

## Results

### Clinicopathological characteristics and surgical outcomes

Baseline clinicopathological characteristics and surgical outcomes are shown in Table 1. The mean patient age was 60.53 (±12.23) years. ER was non-curative in a total of 85 patients (28.1%) for at least two reasons, including lateral or vertical margin involvement combined with LVI. Most patients underwent laparoscopic gastrectomy and the mean operative time was 188.97 ± 73.96 min. After gastrectomy, 44 (14.6%) patients had residual cancer and 26 (9.5%) LNM. In our previous short-term follow up study, there are 16.2% residual cancer and 9.7% LNM^17^. A total of 145 (48%) patients developed postoperative complications and the incidence of major complications was 5.3%. The mean follow-up period after surgery was 41.98 ± 21.23 months, which is longer than 26.7 ± 16.4 months of the previous study.

**Table 1:** Baseline clinicopathological characteristics and surgical outcomes.

Characteristics	Value
Age, (year, mean ± SD)	60.53 ± 12.23
Male, no. (%)	215 (71.2)
BMI, (kg/m^2^, mean ± SD)	23.43 ± 3.18
Comorbidity, no. (%)	168 (55.6)
**ASA score, no. (%)**	
1	146 (48.3)
2	115 (38.1)
3	40 (13.3)
4	1 (0.3)
**Longitudinal location, no. (%)**	
Upper one-third	44 (14.6)
Mid one-third	127 (42.1)
Lower one-third	131 (43.3)
**Cross-sectional location, no. (%)**	
Anterior wall	61 (20.2)
Posterior wall	75 (24.8)
Lesser curvature	100 (33.1)
Greater curvature	66 (21.9)
**Gross type, no. (%)**	
Elevated	130 (43.1)
Flat	78 (25.8)
Depressed	94 (31.1)
**WHO classification, no. (%)**	
Well differentiated	85 (28.1)
Moderately differentiated	125 (41.5)
Poorly differentiated	49 (16.2)
Signet ring cell	39 (12.9)
Carcinoma with lymphoid stroma	4 (1.3)
**Lauren classification, no. (%)**	
Intestinal	221 (73.3)
Diffuse	35 (11.7)
Mixed	25 (8.3)
^ǂ^Unknown	16 (5.4)
Underterminate	4 (1.3)
Tumor size, (cm, mean ± SD)	2.34 ± 1.40
ER specimen size, (cm, mean ± SD)	3.64 ± 1.69
**Cause of additive surgery, no. (%)**	
**Resection margin**	
Lateral margin (+)	34 (11.3)
Vertical margin (+)	66 (21.9)
LVI, no. (%)	65 (21.5)
^*^Others, no. (%)	14 (4.6)
More than two, no. (%)	85 (28.1)
**Operation type, no. (%)**	
Laparoscopic	273 (90.4)
Open	29 (9.6)
**Resection extent, no. (%)**	
Total	47 (15.6)
^Ɨ^Subtotal	255 (84.4)
**Extent of lymphadenectomy, no. (%)**	
D1 + α	16 (5.3)
D1 + β	229 (75.8)
D2	57 (18.9)
Residual cancer, no. (%)	44 (14.6)
Harvested lymph node, (no, mean ± SD)	37.14 ± 15.54
Lymph node metastasis, no. (%)	26 (9.5)
Operation time, (min, mean ± SD)	188.97 ± 73.96
EBL, (cc, mean ± SD)	108.35 ± 295.67
Perioperative transfusion, no. (%)	4 (1.3)
Time to first flatus, (day, mean ± SD)	3.43 ± 0.99
Time to start liquid diet, (day, mean ± SD)	3.48 ± 2.43
Postoperative hospital day, (day, mean ± SD)	8.65 ± 6.59
Time of drain removal, (day, mean ± SD)	3.05 ± 3.49
Maximal postoperative CRP, (mg/L, mean ± SD)	82.69 ± 73.51
Maximal postoperative WBC, (10^3^/µL, mean ± SD)	12.87 ± 3.61
Postoperative overall complication, no. (%)	145 (48.0)
Postoperative major complication, no. (%)	16 (5.3)
Follow-up period, (months, mean ± SD)	41.98 ± 21.23

^*^Others: Poorly differentiated adenocarcinoma with signet ring cell features, Unknown resection margin due to fragmentation.

^†^Two cases of pylorus-preserving gastrectomy are included in the category of subtotal gastrectomy.

^‡^There is no mentioned about pathological report

SD, standard deviation; BMI, body mass index; ASA, American Society of Anesthesiologists; WHO, World Health Organization; ER, Endoscopic resection; LVI, Lymphovascular invasion; CRP, c-reactive protein; WBC, white blood cell; EBL, estimated intraoperative blood loss.

### The relationship between time interval and major complications

To validate the perioperative safety of patients who underwent additional surgery, we analyzed the relationship between the time from ER to additional surgery and the major complications by drawing a receiver-operating characteristic (ROC) curve. The area under the curve (AUC) was 0.579 (95% CI, 0.521–0.635), associated with a sensitivity of 50% and a specificity of 56.7% (Supplemental Fig. 1). These results suggest that the time interval of additional surgery did not affect the incidence of major complications.

### The optimal time interval between ER and additional surgery

We sought correlations between the time to additional surgery and the difficulty of that surgery. The raw data on the time from ER to surgery, and the operative time and EBL, are plotted in Supplemental Fig. 2. The operative time and the EBL decreased significantly as the time interval increased (r = −0292, *P* < 0.001, and r = −0.135, *P* = 0.019, respectively).

**Figure 1:**
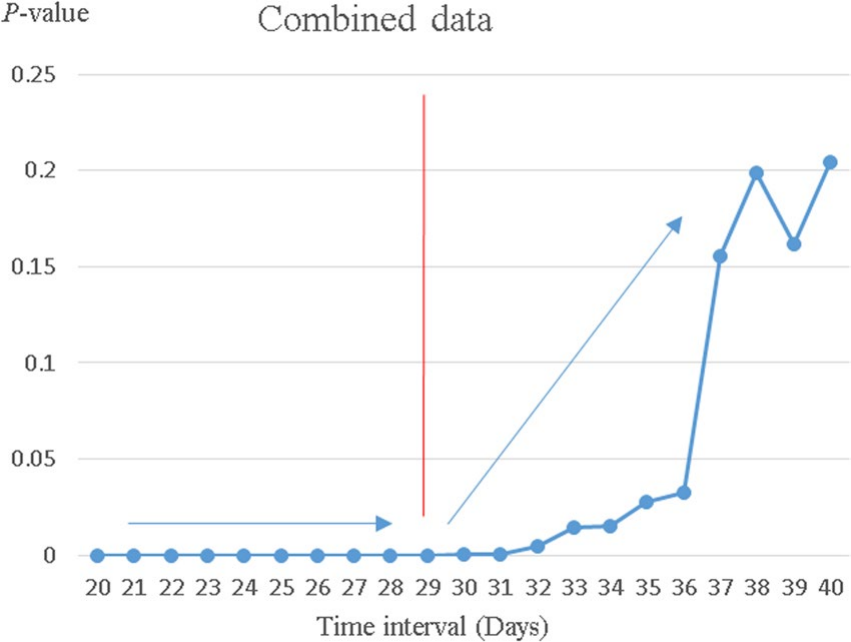
The time intervals since endoscopic resection (days) associated with the greatest differences between the amounts of blood loss and the operative times in the early and later groups were evaluated with the aid of the MANOVA test. The relationship between the time elapsed since endoscopic resection and a combination of operative time and the amount of intraoperative blood loss. The greatest difference and the change of the slope of the graph was around day 29 (the cut-off point), and no significant difference was evident from after day 36.

We next performed one-way MANOVA to validate the optimal time interval (29 days) between non-curative ER and surgery identified in our previous study. In our previous study, we analyzed the correlation between the time interval of additive surgery and the difficulty of surgery by using one- way MANOVA. The time interval point, at which the operative time and the EBL of the earlier operation group and the later operation group showed the greatest disparities. This difference was most pronounced at day 29^17^. In this study, differences between the early and later surgical group were evident over the time interval (25–35 days). The differences were greatest on day 29, at which time the slopes of the graphs changed direction. We thus confirmed that 29 days was the appropriate cut-off point. No significant between-group difference was apparent from after day 36 (Fig. 1, Supplemental Fig. 3).

### Short-term surgical outcomes of both groups

Table 2 compares the two groups by time to surgery after ER. Of the 302 patients, 133 (44.0%) were in the earlier operation group (≤29 days; group A), and 169 (56%) in the later operation group (>29 days; group B). In our previous study, there are 78 (50.6%) were categorized as group A, and 76 (49.4%) as group B^17^. The percentage of patients in group B was more than in our previous study. The two groups did not differ significantly in terms of any of age, sex, body mass index, comorbidities except for tumor size, or ER specimen size. In the later operation group, the operative time, the EBL, the number of perioperative transfusions, the time to drain removal, the drainage volume on postoperative day (POD) 1, the maximal postoperative C-reactive protein (CRP) level, and the duration of postoperative hospital stay were all better than that of the early operation group. The groups did not differ in terms of the numbers of overall or major postoperative complications, or locoregional or distant recurrences.

**Table 2:** Comparison between Two Groups according to Surgery Time after ER

Characteristics	Group A (≤29days) (n = 133)	Group B (>29days) (n = 169)	*P*
Age, (years, mean ± SD)	62.04 ± 9.17	61.14 ± 9.76	0.417
Male, no. (%)	90 (67.7)	125 (74.0)	0.142
BMI, (kg/m^2^, mean ± SD)	23.77 ± 3.12	23.24 ± 2.73	0.117
Comorbidity, no. (%)	68 (51.1)	100 (59.2)	0.162
ASA score, no. (%)			**0.039**
1	73 (54.9)	73 (43.2)	
2	40 (30.1)	75 (44.4)	
3	19 (14.3)	21 (12.4)	
4	1 (0.7)	0 (0.0)	
Tumor size, (cm, mean ± SD)	2.62 ± 1.61	2.20 ± 1.30	**0.011**
ER specimen size, (cm, mean ± SD)	4.10 ± 1.71	3.29 ± 1.56	**<0.001**
Operation type, no. (%)			0.486
Laparoscopic	122 (91.7)	151 (89.3)	
Open	11 (8.3)	18 (10.7)	
Resection extent, no. (%)			0.150
Total	24 (18.0)	23 (13.6)	
Subtotal	109 (82.0)	146 (86.4)	
Type of reconstruction, no. (%)			0.090
Billroth I	74 (55.6)	85 (50.3)	
Billroth II	25 (18.8)	49 (29.0)	
Roux-en-Y	32 (24.1)	35 (20.7)	
Gastro-gastrostomy	2 (1.5)	0 (0.0)	
Extent of lymphadenectomy, no. (%)			**0.019**
D1 (α + β)	111 (83.5)	135 (79.9)	
D2	22 (16.5)	34 (20.1)	
Harvested LN, (no, mean ± SD)	39.86 ± 17.47	34.99 ± 13.49	**0.007**
Existence of metastatic LN, no. (%)			0.292
Yes	14 (10.5)	12 (7.1)	
No	119 (89.5)	157 (92.9)	
Operation time, (min, mean ± SD)	210.67 ± 76.72	173.01 ± 66.07	**<0.001**
EBL, (cc, mean ± SD)	414.95 ± 35.98	135.45 ± 10.42	**0.010**
Intraoperative transfusion, no. (%)			**0.023**
Yes	4 (3.0)	0 (0.0)	
No	129 (97.0)	169 (100)	
Time to first flatus, (day, mean ± SD)	3.45 ± 1.01	3.43 ± 0.93	0.823
Time to start liquid diet, (day, mean ± SD)	3.65 ± 2.13	3.37 ± 2.63	0.330
Time of drain removal, (day, mean ± SD)	4.20 ± 4.19	3.36 ± 3.29	**0.050**
POD#1 drain discharge, (cc, mean ± SD)	159.92 ± 272.72	94.53 ± 144.84	**0.008**
POD#2 drain discharge, (cc, mean ± SD)	98.74 ± 123.79	83.73 ± 132.86	0.321
Maximal postoperative CRP, (mg/L, mean ± SD)	96.48 ± 73.05	74.40 ± 72.41	**0.009**
Maximal postoperative WBC, (10^3^/µL, mean ± SD)	13.21 ± 3.49	12.79 ± 3.56	0.211
Postoperative overall complication, no. (%)	66 (49.6)	79 (46.7)	0.619
Postoperative major complication, no. (%)	10 (7.5)	6 (3.6)	0.126
Postoperative hospital day, (day, mean ± SD)	10.05 ± 8.17	7.60 ± 4.74	**0.001**
Follow-up period, (months, mean ± SD)	37.02 ± 20.54	44.18 ± 19.49	**0.002**
Locoregional recurrence, no. (%)	2 (1.5)	2 (1.2)	0.784
Distant recurrence, no. (%)	4 (3.0)	2 (1.2)	0.409

SD, standard deviation; BMI, body mass index; ASA, American Society of Anesthesiologists; LN, Lymph node; ER, Endoscopic Resection; EBL, Estimated blood loss; CRP, c-reactive protein; WBC, white blood cell; POD, Post-operative day; EBL, estimated intraoperative blood loss.

Supplemental Fig. 4 shows the data on perioperative surgical outcomes, including the CRP levels, the white blood cell (WBC) counts, and drainage volumes. There was no significant between-group difference except in terms of the POD 1 drainage volume, as mentioned above.

### Long-term oncological outcomes of both groups

The median follow-up times after surgery were 37.02 ± 20.54 and 44.18 ± 19.49 months in the early operation and later operation groups, respectively (*P* = 0.002). During the follow-up period, six patients of the early operation group and four of the later operation group experienced recurrences. In the early operation group, distant metastasis occurred in four patients and locoregional recurrence in two. The former four patients died; their characteristics are described in Supplementary Table 1. The median survival time was estimated to be 58.6 months in the Group A (≤29days) and 62.7 months in the Group B (>29days). In the all patients, the median survival time was 61.8 months. The 5-year survival rates were as follows: Group A (≤29days): 92%, Group B (>29days): 99%. There was no significant difference in the 5-year survival rate of two groups.

**Figure 2:**
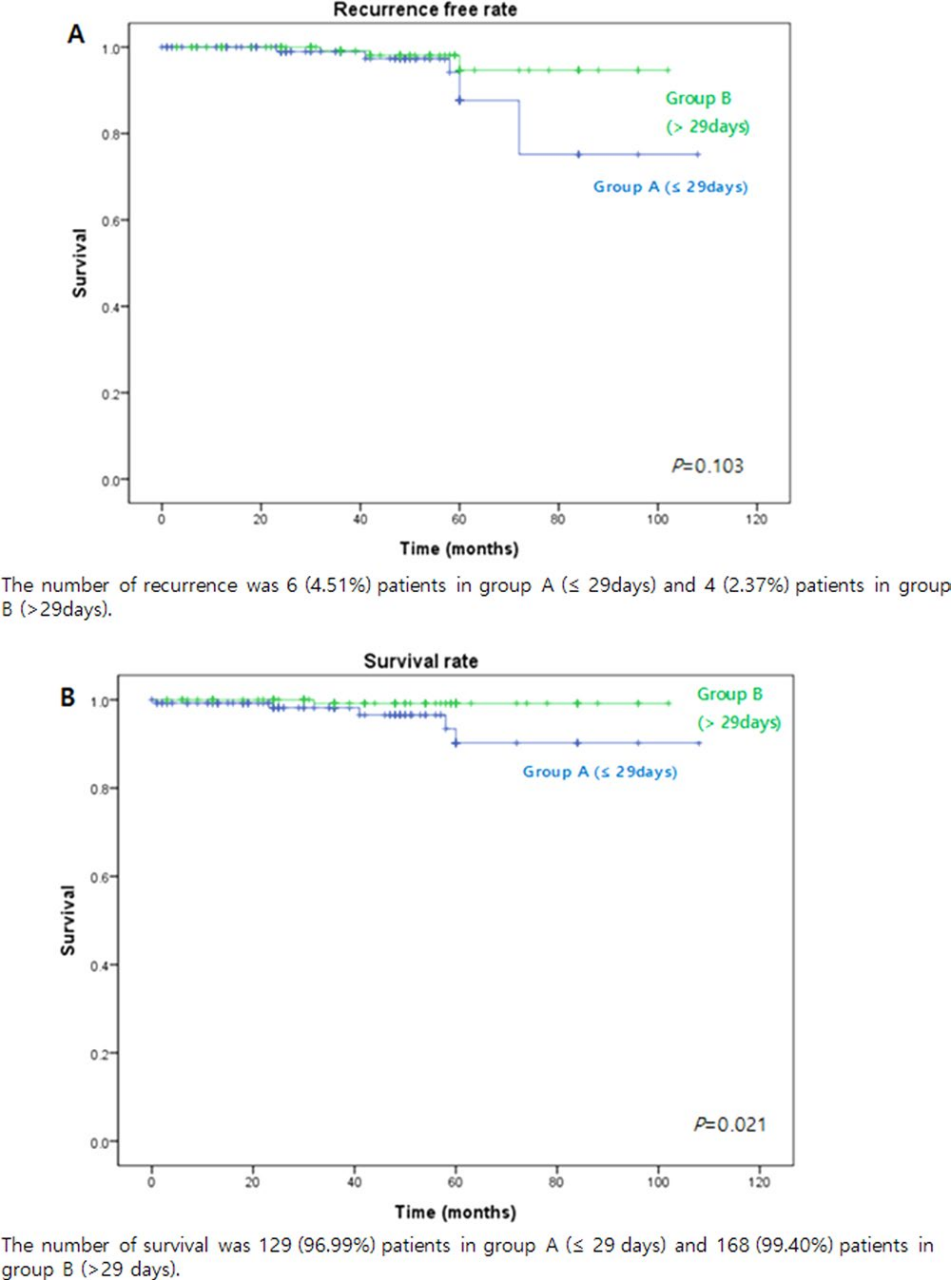
Kaplan–Meier plots of overall recurrence and survival. (**A**) The recurrence-free rate curve of those for whom the interval between endoscopic resection (ER) and additional surgery was 29 days. (**B**) The survival rate curve of such patients. The recurrence-free rate did not differ after the later operation, but the survival rate did.

The recurrence rates did not differ significantly between the groups. Interestingly, the survival rate was better in the later operation group than the early group (Fig. 2). Of factors possibly affecting recurrence and survival, LNM was significant in both univariate and multivariate analyses (Tables 3 and 4; Supplementary Fig. 5).

**Table 3:** Univariable and Multivariable analysis according to Recurrence.

Univariable			Multivariable	
Factor	HR (95% CI)	*P*	HR (95% CI)	*P*
Age	1.010 (0.945–1.079)	0.768		
Sex		0.677		
Male	Reference			
Female	0.719 (0.152–3.405)			
Time interval, day		0.121		
<29	Reference			
≥29	0.367 (0.103–1.302)			
Tumor location				
Upper	Reference			
Middle	0.311 (0.059–1.636)	0.168		
Lower	0.421 (0.089–1.997)	0.276		
Tumor size (cm)				
<2	Reference			
≥2	0.610 (0.169–2.206)	0.451		
T Stage				
No residual tumor	Reference			
T1a, T1b	0.819 (0.102–6.559)	0.851		
T2a, T3	4.852 (0.597–39.408)	0.139		
LNM		**0.001**		**0.010**
No	Reference		Reference	
Yes	8.678 (2.439–30.859)		6.061 (1.538–23.884)	
LVI		**0.036**		0.197
No	Reference		Reference	
Yes	3.933 (1.095–14.129)		2.500 (0.621–10.057)	

LVI, Lymphovascular invasion; LNM, Lymph node metastasis; CI, confidence interval; HR, hazards ratio.

Lymphovascular invasion (LVI) and Lymph node metastasis (LNM) are included in the multivariable model.

**Table 4:** Univariable and Multivariable analysis according to Survival.

Univariable			Multivariable	
Factor	HR (95% CI)	*P*	HR (95% CI)	*P*
Age	1.024 (0.938–1.118)	0.592		
Sex		0.556		
Male	Reference			
Female	0.524 (0.061–4.494)			
Time interval, day		0.053		0.103
<29	Reference		Reference	
≥29	0.120 (0.014–1.0)		0.193 (0.027–1.394)	
Tumor location				
Upper	Reference			
Middle	1.040 (0.105–10.269)	0.973		
Lower	0.667 (0.059–7.53)	0.743		
Tumor size (cm)				
<2	Reference			
≥2	0.928 (0.184–4.637)	0.928		
T Stage				
No residual tumor	Reference			
§T1a, T1b	0.590 (0.023–14.992)	0.749		
T2a, T3	13.904 (1.813–106.607)	**0.011**	1.202 (0.105–1.394)	0.883
LNM		**0.002**		**<0.001**
NO	Reference		Reference	
YES	17.870 (2.841–112.414)		11.575 (1.829–73.237)	
LVI		0.101		
NO	Reference			
YES	4.200 (0.755–23.354)			

LVI, Lymphovascular invasion; LNM, Lymph node metastasis; CI, confidence interval; HR, hazards ratio.

^§^Firth corrected cox regression performed for sparse data (T stage)

Time interval, T stage and Lymph node metastasis (LNM) are included in the multivariable model.

## Discussion

In this long-term follow-up study, we found that additional surgery performed about 1 month after ER afforded better short-term surgical outcomes and long-term oncological safety. Minimally invasive surgery for the treatment of EGC has increased in popularity, and many reports have addressed learning curve effects in terms of laparoscopy-assisted gastrectomy; operative time, extent of intraoperative bleeding, and postoperative complication rate fell with increasing surgical experience^20–22^. Another study found that a longer operation time, more bleeding, and more frequent transfusion were all associated with more challenging and difficult operations^23^. We evaluated perioperative patient safety using the Clavien–Dindo system; such safety is the highest priority when planning surgery^24–26^. We found no significant relationship between the major complication rate and the time to surgery after ER. This means that there is no relationship between the additional surgery time interval after ER and the important complications that may occur in the patient.

Second to surgical complications, the operative time, and EBL would be applied as next endpoints to estimate operative feasibility. In these respects, we evaluated the association between time interval of additioanl surgery after non-curative ER and the two factors, operative time and EBL. As shown in the results, these parameters tended to decrease as the time interval increased. In our previous study, we used the MANOVA test to define the time from ER at which the later operative time and the EBL differed maximally between an early and later operation group; we employed the same method here. The greatest differences (reflected by changes in the slopes of the graphs) were evident 29 days after ER. Many studies have reported that ER-induced ulcers are usually in the healing or scarring stage 4–8 weeks after ER^27–30^. ER-induced inflammation and the lack of ulcer healing may render early operation (within 4 weeks) more difficult than later operation. Also, the postoperative hospital stay was significantly shorter in the later operative group. In recent years, the length of stay has been emphasized not only as an indicator of healthcare costs, but also because it is closely related to complications associated with surgery, and surgical outcomes^31–33^. Between-group differences were evident in terms of the WBC counts, CRP levels, and drainage volumes; these are all markers of surgical trauma^34,35^. Therefore, our long-term follow-up study validated our earlier suggestion that the optimal time interval from ER to additional surgery was about 1 month.

We performed subgroup analysis by surgical experience (Supplementary Table 2). We defined a group of experienced surgeons in previous studies as surgeons with more than five years of experience in gastric surgery. Group A was 46.6% (62/133) and Group B was 65.7% (111/169). Because the experienced surgeon rate of the earlier operation group (≤29days; group A) is lower than the later operation group (>29 days; group B), we performed subgroup analysis for patients in the experienced surgeon group in order to correct the surgeon specific variable factor. On subgroup analysis in the experienced surgeon group, the operative time and postoperative hospital stay of patients in the early operation group were significantly longer than in the later group. In the late group, the EBL was significantly lower than in the early operation group. But there is no difference recurrence between two groups.

In this long-term follow-up study, we also evaluated oncological outcomes in terms of the optimal timing of surgery. As mentioned above, during follow-up, 10 patients experienced locoregional or distant recurrences, of whom 6 were in the early and 4 in the later operation group. The recurrence incidence did not differ between the two groups, but the survival rate did; more patients operated upon early rather than late developed LNM, suggesting that the biological behavior of the cancer was prognostically more important than the time between ER and surgery. Recently, the requirement for perioperative blood transfusion and the extent of intraoperative blood loss have been suggested to be potentially (negatively) prognostic in terms of long-term outcomes^36–38^. This may be why the survival rate of our early operation group was poorer than that of the later group.

The limitations of our study include the retrospective design of the work and the inclusion of patients treated in only two tertiary centers. Additional prospective multicenter studies are needed to validate our findings. However, we validated our earlier study on the optimal timing of additional gastrectomy after non-curative ER, and our work may be of assistance to other surgeons.

In conclusion, we suggest that the interval between non-curative ER and additional gastrectomy should be about 1 month. This was associated with better surgical outcomes and oncological safety than earlier surgery.

## Methods

### Study population

We retrospectively collected data on patients diagnosed with EGC who underwent ER at the Severance and Gangnam Severance Hospitals, Yonsei University College of Medicine, Seoul, Korea, between January 2007 and December 2014. A total of 2,743 such patients were enrolled. Of these, 330 (12.0%) underwent non-curative ER as revealed histologically, and therefore required additional surgery. We excluded patients with any other malignancies, those who underwent combined operations, those who underwent emergency operations to treat ER complications (such as perforation or bleeding), and those who died because of other malignancies. We performed long-term follow-up on 154 patients enrolled in our previous study and a further 148 patients. Thus, in total, we analyzed 302 patients who underwent additional gastrectomy after non-curative ER.

The indications for ER included the expanded criteria: (1) a differentiated intramucosal adenocarcinoma ≤3 cm in diameter, without lymphovascular invasion (LVI), irrespective of ulceration status; (2) a differentiated intramucosal adenocarcinoma without LVI or ulceration, irrespective of tumor diameter; (3) an undifferentiated intramucosal cancer ≤2 cm in diameter, without LVI or ulceration; and, (4) a differentiated adenocarcinoma ≤ 3 cm in diameter exhibiting minimal submucosal invasion, without LVI^7,39^. EGC patients who underwent non-curative ER included those with incomplete margin resections, or LVI, or who otherwise did not fall within (exceeded) the expanded ER criteria^39^.

We analyzed clinicopathological characteristics, comorbidities, American Society of Anesthesiologists (ASA) scores, surgical outcomes (operative time and estimated intraoperative blood loss [EBL]), postoperative complications, and oncological outcomes (locoregional and distant recurrences). Postoperative complications were graded using the Clavien–Dindo classification; complications of grade ≥III were defined as ‘major,’ being potentially life-threatening^25,40^.

The patients were divided into two groups depending on the time interval (a cutoff between 1 and 60 days; please see below) between ER and surgery. Then, the surgical outcomes of the earlier operation group (group A) and the later operation group (group B) were compared to identify the optimal time interval from non-curative ER to surgery.

Informed consent was obtained from all patients before the procedures. This study was approved by the Institutional Review Board of Yonsei University College of Medicine and was conducted in accordance with the ethical principles of the Declaration of Helsinki (IRB No. 3-2018-0022).

### Statistical analysis

Categorical variables are presented as numbers with percentages and were compared using the chi- squared or Fisher’s exact test. Continuous variables are presented as means ± standard deviations and were compared with the aid of Student’s *t*-test. A *P* value <0.05 was considered to reflect statistical significance. The relationship between the time interval from ER to surgery, and major complications, was evaluated by calculating the area under the receiver operating characteristic (AUROC) curve. Multivariate analyses of variance (MANOVAs) were used to explore the effects of the time interval between non-curative ER and additional surgery on the later operative time and the EBL. When data points lay >1.5-fold of the interquartile range (IQR) above the third or below the first quartile (outliers), we treated them as missing when calculating operative times and EBLs. The recurrence-free and overall survival rates of the two groups were calculated using the Kaplan–Meier method. To identify risk factors for recurrence and survival after additional surgery, we performed both univariate and multivariate logistic regression analyses. Cox’s regression hazard model was used for the multivariable analysis. We included only those variables that exhibited *P* values <0.1 on univariate analysis in the multivariable analysis. All statistical calculations were performed with the aid of SPSS version 23.0 (SPSS Inc., Chicago, IL, USA) and SAS MANOVA version 9.3 (SAS Inc., Cary, NC, USA).

## Supplementary information

Supplementary Table 1. The characteristics of patients with distant recurrence in Group A (≤ 29days) and Group B (>29days)
