# Supplementary material for: The optimal timing of additional surgery after non-curative endoscopic resection to treat early gastric cancer: long-term follow-up study
Source: Sci Rep. 2019 Dec 4;9:18331. doi: 10.1038/s41598-019-54778-8 (PMC6892792; doi:10.1038/s41598-019-54778-8)
Supplement: Supplementary file 1 — Supplementary Table 1. The characteristics of patients with distant recurrence in Group A (≤ 29days) and Group B (>29days) [file 41598_2019_54778_MOESM1_ESM.docx]

Title page

**Original article**

**The optimal timing of additional surgery after non-curative endoscopic resection to treat early gastric cancer: long-term follow-up study**

^1, 5^Jae Hwang Cha, MD,^1,3^Jie-Hyun Kim, MD, PhD, ^4^Hyoung-Il Kim, MD, ^1,3^Da Hyun Jung, MD, ^1,3^Jae Jun Park, MD, PhD, ^1,3^Young Hoon Youn, MD, PhD, ^1,3^Hyojin Park, MD, PhD, ^2,3^Seung Ho Choi, MD, PhD, ^4^Jae-Ho Cheong, MD, PhD, ^4^Woo Jin Hyung, MD, PhD, ^4^Sung Hoon Noh, MD, PhD

^1^Department of Internal Medicine, ^2^Department of Surgery,

^3^Gangnam Severance Hospital, Yonsei University College of Medicine, Seoul, Korea

^4^Department of Surgery, Severance Hospital, Yonsei University College of Medicine, Seoul, Korea

^5^Dong-A University College of Medicine, Busan, Republic of Korea

**1. Corresponding author**: Jie-Hyun Kim, MD, PhD

**Address:** Department of Internal Medicine, Gangnam Severance Hospital, Yonsei University College of Medicine, 211 Eonjuro, Gangnam-gu, Seoul, Korea, 135-720

**Phone:** 82-2-2019-3505

**Fax:** 82-2-3463-3882

**E-mail:** OTILIA94@yuhs.ac

**2. Corresponding author**: Hyoung-Il Kim, MD

**Address:** Department of Surgery Yonsei University College of Medicine, Ludlow faculty research building #220, 50 Yonsei-ro, Seodaemun-gu, Seoul, Korea 120-752

**Phone:** 82-2-2228-2100

**Fax:** 82-2-313-8289

**E-mail:** CAIRUS@yuhs.ac

Supplementary Table 1. The characteristics of patients with distant recurrence in Group A (≤ 29days)

and Group B (>29days)

| Distant recurrence (Group A) | | | | |
| --- | --- | --- | --- | --- |
| Age/Sex | 52/Male | 57/Female | 56/Male | 58/Male |
| Tumor location | Angle/PW | Antrum/AW | UB/GC | Antrum/GC |
| Size (㎝) | 1.4 | 3.2 | 3.3 | 0.9 |
| Differentiation | MD/Intestinal | SRC | MD/Intestinal | SRC |
| Operation type | LDG | LDG | LTG | LDG |
| Non-curative resection | LVI (+) / VRM (+) | LVI (-)/VRM (+) | LVI (+) /VRM (+) | LVI (+) / VRM (+) |
| Time interval after ER (day) | 27 | 20 | 8 | 8 |
| Residual T stage | pT3 | (-) | (-) | (-) |
| LN metastasis | 0/38 | 0/38 | 1/20 | 2/24 |
| Postoperative major complication | (-) | (-) | (-) | (-) |
| Postoperative follow up duration (months) | Expire, 41 | Expire, 65 | Expire, 33 | Expire, 33 |
| Location of metastasis | Liver S4, Peritoneal carcinomatosis | Peritoneal carcinomatosis | Left adrenal gland | Both lung |

PW, posterior wall; AW, anterior wall; UB, upper body; GC, greater curvature; MD, moderately differentiated; PD, poorly differentiated; SRC, signet ring cell carcinoma; LVI, lymphovascular invasion; VRM, vertical resection margin; LRM, lateral resection margin; LN, lymph node; LDG, laparoscopic distal gastrectomy; LTG, laparoscopic total gastrectomy

| Distant recurrence (Group B) | | |
| --- | --- | --- |
| Age/Sex | 73/M | 48/F |
| Tumor location | MB/GC | UB/AW |
| Size (㎝) | 1.2 | 2 |
| Differentiation | PD/Intestinal | SRC |
| Operation type | LDG | LTG |
| Non-curative resection | LVI (+) / LRM (+) / VRM (+) | LVI (+) / LRM (+) / VRM (+) |
| Time interval after ER (day) | 100 | 37 |
| Residual T stage | (-) | pT1a |
| LN metastasis | 0/15 | 18/54 |
| Postoperative major complication | (+) | (+) |
| Postoperative follow up duration (months) | Expire, 35 | Survive, 110 |
| Location of metastasis | Liver | Bone metastasis |

AW, anterior wall; MB, mid body; UB, upper body; GC, greater curvature; PD, poorly differentiated; SRC, signet ring cell carcinoma; LVI, lymphovascular invasion; VRM, vertical resection margin; LRM, lateral resection margin; LN, lymph node; LDG, laparoscopic distal gastrectomy; LTG, laparoscopic total gastrectomy

Supplementary Table 2. Comparison between two groups according to surgery time after ER in experienced surgeons on surgical outcomes

| Characteristics | Group A (≤ 29days) n=62 | Group B (> 29days) n=111 | *P-*value |
| --- | --- | --- | --- |
| Operation type (n, %) |  |  | 0.888 |
| Laparoscopic | 53 (85.5) | 94 (84.7) |  |
| Open | 9 (14.5) | 17 (15.3) |  |
| Resection extent (n, %) |  |  | 0.406 |
| Total | 11 (17.7) | 20 (18.0) |  |
| Subtotal | 51 (82.3) | 91 (82.0) |  |
| Type of reconstruction (n, %) |  |  | 0.475 |
| Billorth I | 30 (48.4) | 53 (47.7) |  |
| Billorth II | 17 (27.4) | 37 (33.3) |  |
| Roux-en-Y | 14 (22.6) | 21 (18.9) |  |
| Extent of lymphadenectomy (n, %) |  |  | 0.099 |
| D1 (α+β) | 52 (83.9) | 89 (80.2) |  |
| D2 | 10 (16.1) | 22 (18.9) |  |
| Residual cancer (n, %) | 10 (16.1) | 17(15.3) | 0.898 |
| Harvested LN (mean±SD) | 38.02±16.30 | 34.88±14.28 | 0.190 |
| Lymph node metastasis (n, %) | 6 (9.7) | 5 (4.5) | 0.181 |
| Operation time (min, mean±SD) | 199.52±64.47 | 173.78±66.05 | 0.014 |
| Estimated intraop. blood loss (cc, mean±SD) | 113.65±155.82 | 65.59±141.78 | 0.045 |
| Intraop. Transfusion (n, %) | 1 (1.6) | 0 (0.0) | 0.180 |
| Time to first flatus (day, mean±SD) | 3.45±1.04 | 3.42±1.01 | 0.861 |
| Time to start liquid diet (day, mean±SD) | 3.68±1.78 | 3.28±3.17 | 0.364 |
| Postop. Hospital day (day, mean±SD) | 9.87±5.87 | 7.60±5.60 | 0.013 |
| Time of HV removal (day, mean±SD) | 2.37±2.28 | 2.39±3.46 | 0.973 |
| POD#1 HV discharge (cc, mean±SD) | 102.42±117.08 | 79.14±154.07 | 0.302 |
| POD#2 HV discharge (cc, mean±SD) | 50.86±77.82 | 65.95±141.13 | 0.451 |
| Maximal postop. CRP (mg/L, mean±SD) | 84.16±81.18 | 63.64±75.85 | 0.098 |
| Maximal postop. WBC (10^3/µL, mean±SD) | 12.82±3.41 | 12.55±3.34 | 0.610 |
| Postop. overall complication (n, %) | 37 (59.7) | 51 (45.9) | 0.085 |
| Postop. major complication (n, %) | 3 (4.8) | 6 (5.4) | 0.872 |
| F/u duration (months, mean±SD) | 41.10±21.42 | 48.90±20.15 | 0.018 |
| Locoregional recurrence (n, %) | 1 (1.6) | 2 (1.8) | 0.992 |
| Distant recurrence (n, %) | 1 (1.6) | 2 (1.8) |  |

SD, standard deviation; LN, Lymph node; ER, Endoscopic Resection; EBL, Estimated blood loss; CRP, c-reactive protein; WBC, white blood cell; POD, Post-operative day; EBL, estimated intraoperative blood loss


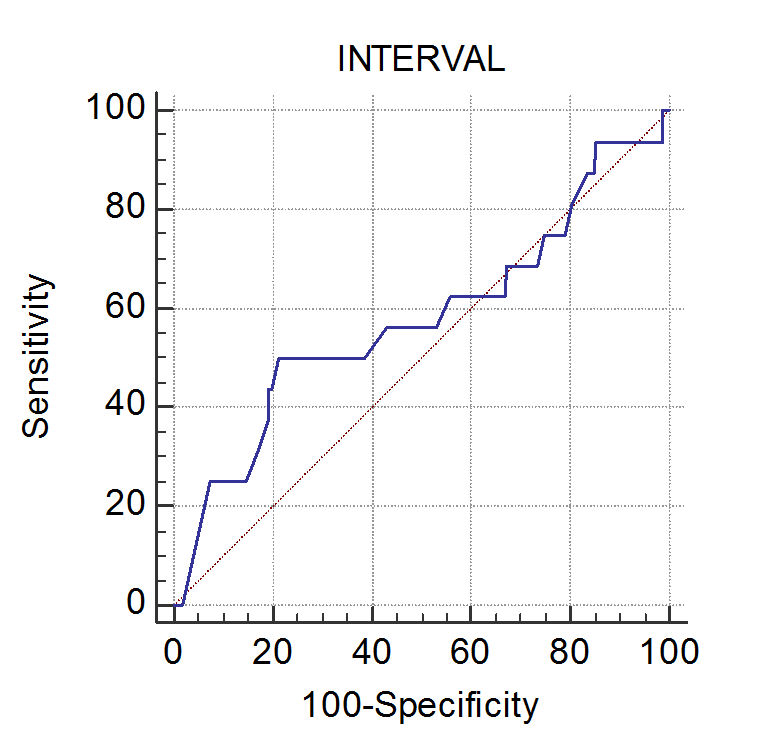


Supplemental Figure 1. A receiver-operating characteristic (ROC) curve drawn by reference to the time interval from endoscopic resection (ER) to additional gastrectomy, and the major complications. No significant relationship is evident between that time interval and the short-term surgical outcomes (AUROC: 0.579; sensitivity: 50%; specificity: 78.67%).


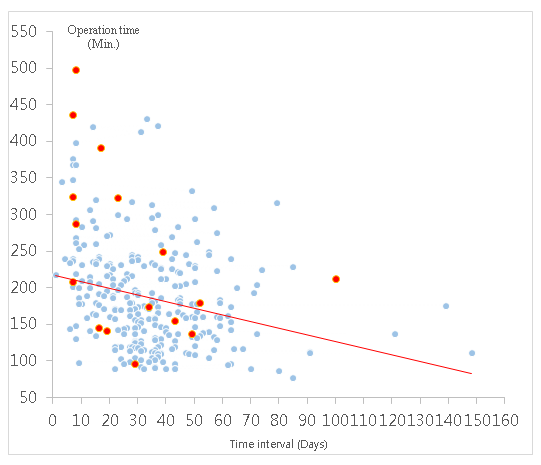


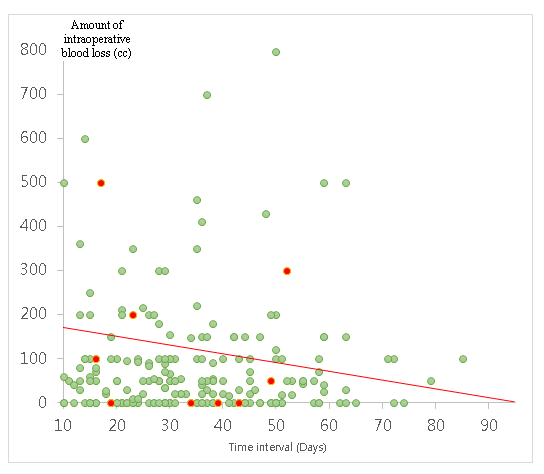


Supplemental Figure 2. A graph of the raw data relevant to any relationship between the operative time and the time elapsed since endoscopic resection (r = −0.292; *P* < 0.001). A graph of the raw data relevant to any relationship between the amount of intraoperative blood loss and the time elapsed since endoscopic resection (r = −0.135; *P* = 0.019). The red dots indicate patients with major complications (Clavien–Dindo grade ≥ III).


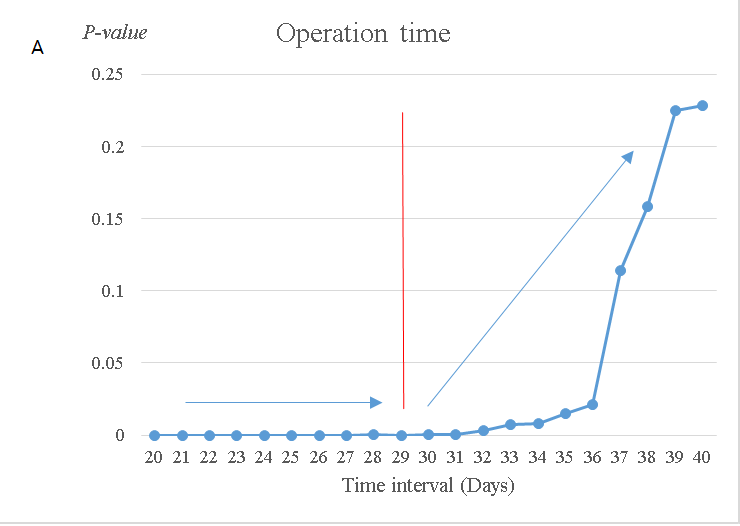


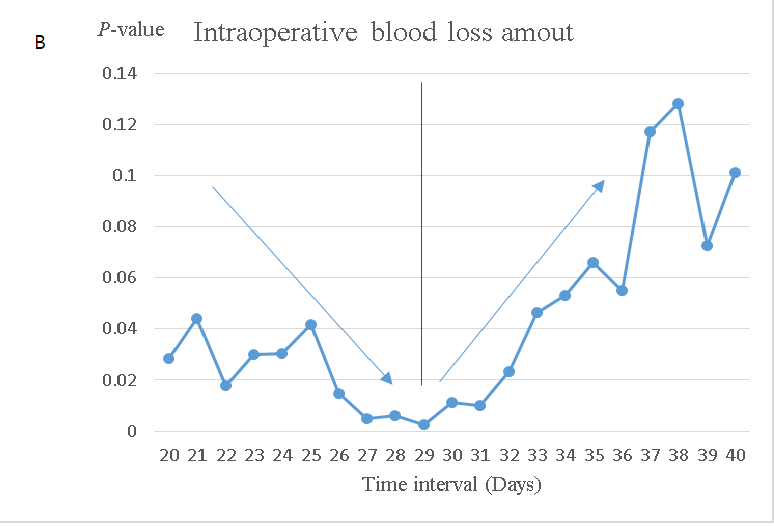


Supplemental Figure 3. The time intervals since endoscopic resection (days) associated with the greatest differences between the amounts of blood loss and the operative times in the early and later groups were evaluated with the aid of the MANOVA test. (A) The relationship between operative time and the time elapsed since endoscopic resection; (B) The relationship between the amount of intraoperative blood loss and the time elapsed since endoscopic resection.


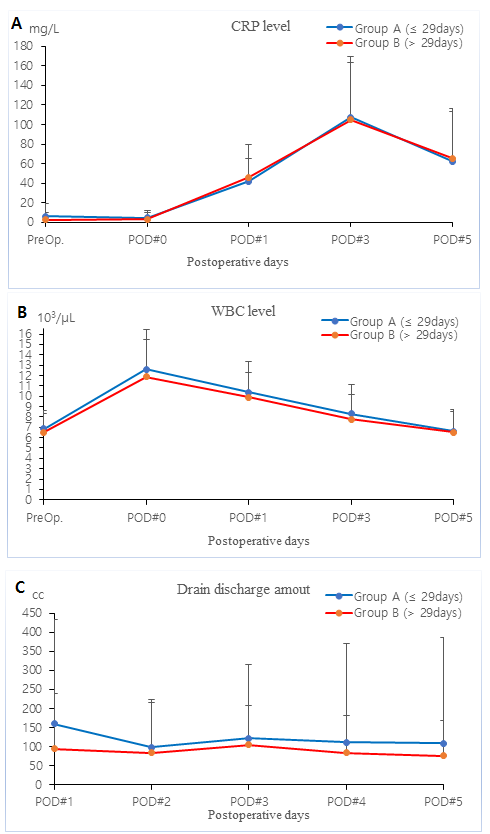


Supplemental Figure 4. Line graph relevant to evaluation of perioperative surgical outcomes in both groups (A: CRP level; B: WBC count, C: drain discharge volume). No significant between-group difference was apparent except in terms of drain discharge volume on POD 1 (P = 0.008).


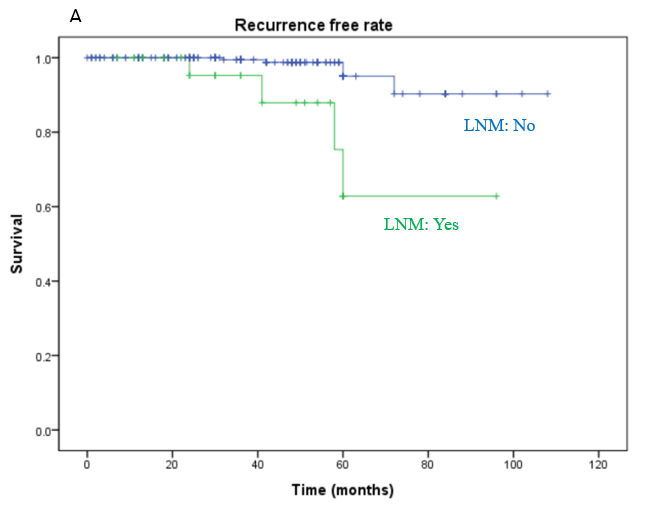


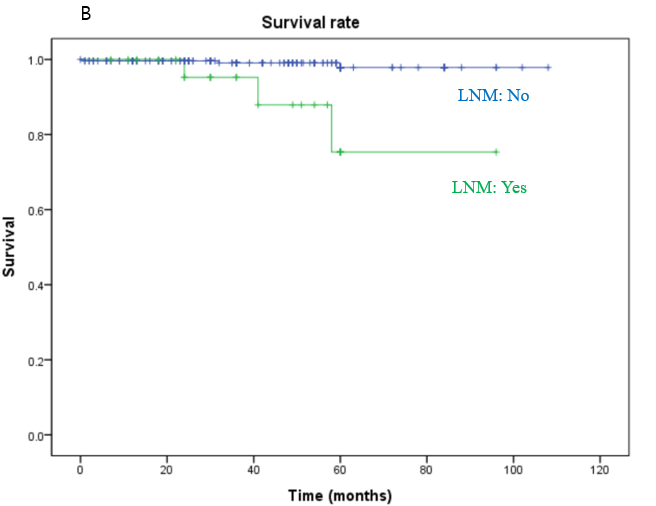


Supplemental Figure 5. Kaplan-Meier plot for overall recurrence and survival association with lymph node metastasis (LNM)

1. Recurrence free rate curve associated with LNM (*P* <0.001).
2. Survival rate curve associated with LNM (*P* <0.001).
